# Prerequisites for the acquisition of mammalian pathogenicity by influenza A virus with a prototypic avian PB2 gene

**DOI:** 10.1038/s41598-017-09560-z

**Published:** 2017-08-31

**Authors:** Chung-Young Lee, Se-Hee An, Ilhwan Kim, Du-Min Go, Dae-Yong Kim, Jun-Gu Choi, Youn-Jeong Lee, Jae-Hong Kim, Hyuk-Joon Kwon

**Affiliations:** 1 Laboratory of Avian Diseases, College of Veterinary Medicine, Seoul National University, 08826 Seoul, Republic of Korea; 2 Laboratory of Poultry Production Medicine, College of Veterinary Medicine, Seoul National University, 08826 Seoul, Republic of Korea; 3 Research Institute for Veterinary Science, College of Veterinary Medicine, Seoul National University, 08826 Seoul, Republic of Korea; 4 Division of Antimicrobial Resistance, Center for Infectious Diseases, National Research Institute of Health, KCDC, Cheongju, Republic of Korea; 5 Department of Veterinary Pathology, College of Veterinary Medicine, Seoul National University, 08826 Seoul, Republic of Korea; 6 Farm Animal Clinical Training and Research Center (FACTRC), GBST, Seoul National University, Kangwon-do, Republic of Korea; 7 Avian Disease Division, Animal and Plant Quarantine Agency, 177, Hyeoksin 8-ro, Gyeongsangbuk-do, 39660 Republic of Korea

## Abstract

The polymerase of avian influenza A virus (AIV) is a heterotrimer composed of PB2, PB1, and PA. PB2 plays a role in overcoming the host barrier; however, the genetic prerequisites for avian PB2 to acquire mammalian pathogenic mutations have not been well elucidated. Previously, we identified a prototypic avian PB2 that conferred non-replicative and non-pathogenic traits to a PR8-derived recombinant virus when it was used to infect mice. Here, we demonstrated that key amino acid mutations (I66M, I109V, and I133V, collectively referred to as MVV) of this prototypic avian PB2 increase the replication efficiency of recombinant PR8 virus carrying the mutated PB2 in both avian and mammalian hosts. The MVV mutations caused no weight loss in mice, but they did allow replication in infected lungs, and the viruses acquired fatal mammalian pathogenic mutations such as Q591R/K, E627K, or D701N in the infected lungs. The MVV mutations are located at the interfaces of the trimer and are predicted to increase the strength of this structure. Thus, gaining MVV mutations might be the first step for AIV to acquire mammalian pathogenicity. These results provide new insights into the evolution of AIV in birds and mammals.

## Introduction

Aquatic birds are natural reservoirs for influenza A virus (IAV), and 16 hemagglutinin (HA) and 9 neuraminidase (NA) subtypes for IAV have been identified^1, 2^. Recently, the additional subtypes H17N10 and H18N11 were discovered in New World bats, which are considered another reservoir for a diverse pool of IAVs^3, 4^. Pigs have long served as intermediate hosts for the reassortment of avian and mammalian IAVs, but direct transmission of avian IAVs to humans has become a worldwide public health threat^2, 5^.

The polymerase of avian influenza virus (AIV) is a heterotrimer composed of PB1, PB2, and PA. In this structure, PA and PB2 bind to the N- and C-termini of PB1, respectively^6, 7^. PB1 functions as an RNA-dependent RNA polymerase, and PA cleaves the cap containing 10-13 nucleotides from host pre-mRNA, which is then captured by PB2^8, 9^. These three subunits determine the host range, tissue tropism and mammalian pathogenicity of AIV^6, 10–13^. Diverse mutations in the polymerase subunits that determine the mammalian pathogenicity of AIV have been reported, and E627K in PB2 is considered a key mutation^10, 13–15^. Amino acid position 627 on PB2 is located in the C-terminal RNA-binding domain, and the E627K mutation is known to increase both RNA binding and polymerase activity, increasing viral replication efficiency at 33 °C, the approximate temperature of the human upper respiratory tract^16, 17^. Furthermore, the E627K mutation may increase the mammalian pathogenicity of AIV by promoting a stronger interaction with mammalian importin-α isoforms and enhancing the NP-PB2 interaction in mammalian cells^18–21^.

We previously identified avian polymerase genes with different degrees of mammalian pathogenicity. A prototypic PB2 gene of an H9N2 low-pathogenic AIV (LPAIV) strain, A/chicken/Korea/01310/2001 (H9N2) (01310), was not replicative and non-pathogenic (no body weight loss) in BALB/c mice after inoculation of 7 + 1 PR8-derived recombinant virus. Furthermore, the PB2 gene of the H9N2 LPAIV strain A/Korea/KBNP-0028/2000 (H9N2) (0028) was replicative but non-pathogenic in BALB/c mice, and we identified candidate amino acids related to the replication of 0028 PB2 in mouse lungs by comparing the amino acid sequences of PB2 proteins^22^. Importantly, the identification of key amino acids in 0028 PB2 that confer replicative ability, but not pathogenicity, may improve understanding of the first-step mutations that occur in prototypic 01310 PB2 to facilitate the acquisition of fatal mammalian pathogenicity. Here, we identified key mutations (I66M, I109V, and I133V, collectively referred to as “MVV”) that increase replication efficiency in both avian and mammalian hosts and are predicted to increase the structural integrity of the trimeric polymerase. These MVV mutations are essential prerequisites for the subsequent acquisition of fatal mammalian pathogenic mutations. Collectively, these results provide important insights into the first evolutionary step taken by AIV to acquire pathogenicity in mammals.

## Results

### Identification of key amino acid mutations

**Figure 1:**
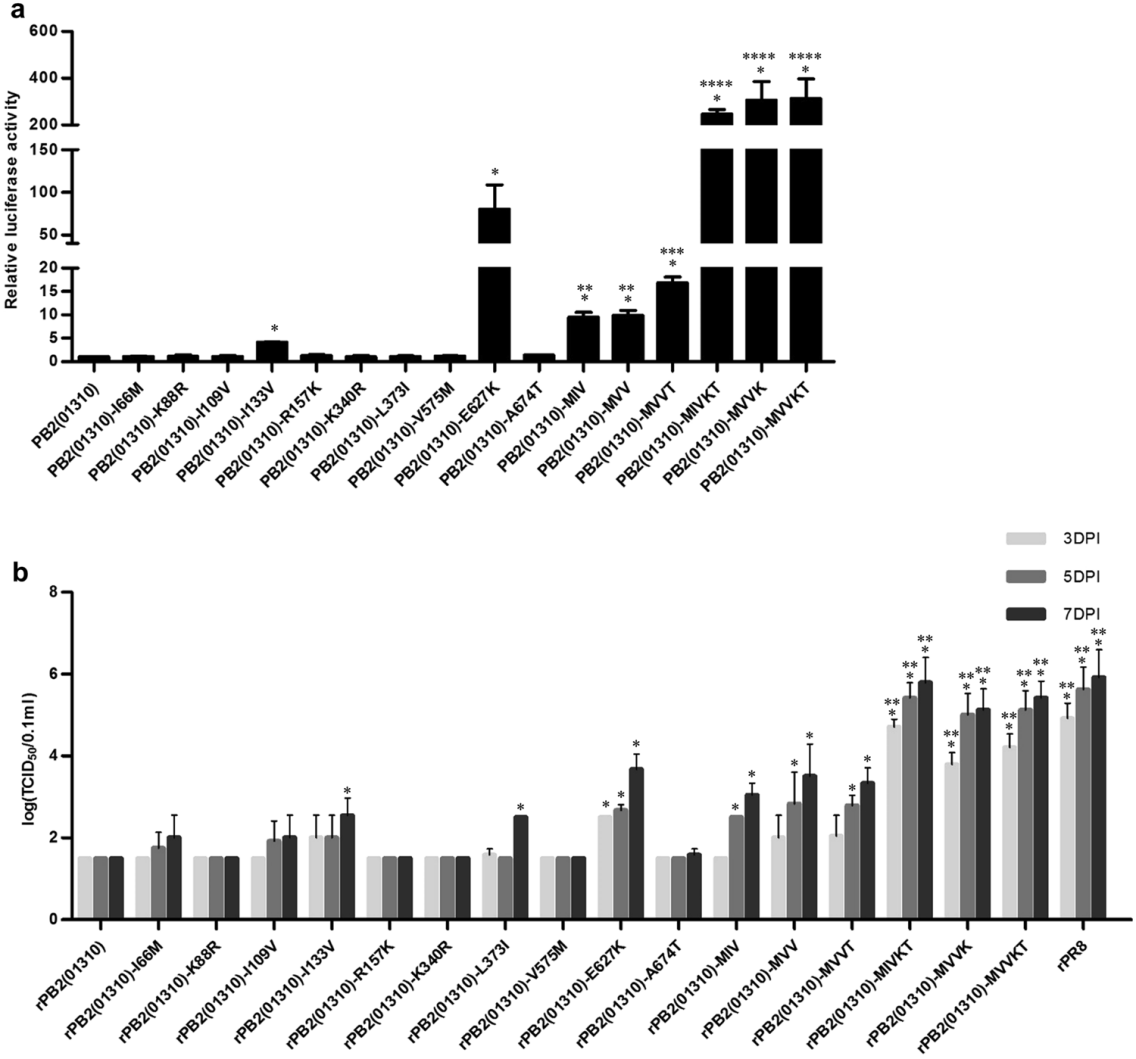
Viral polymerase activity and growth kinetics of 01310 PB2 variants. **(a**) Viral polymerase activity was measured using mini-genome assays in 293T cells. The data were normalized to the polymerase activity of the wild-type 01310 PB2 gene. Statistical significance was calculated using Student’s t-test (compared to PB2(01310), *P < 0.05; compared to PB2(01310)-I133V, **P < 0.05; compared to PB2(01310)-MVV, ***P < 0.05). (**b**) Replication efficiency of 01310 PB2 variants in MDCK cells at 37 °C. Wild-type rPB2(01310) and mutant viruses were used to infect MDCK cells at 10^7^ EID_50_/0.1 ml, and the TCID_50_ was determined at 3, 5, and 7 dpi. Statistical significance was analysed by two-way analysis of variance with Bonferroni post-test correction (compared to rPB2(01310), *P < 0.05; compared to rPB2(01310)-E627K, **P < 0.05). The data presented are the average of three independent experiments ± s.d. from one experiment.

Based on previously identified candidate amino acids, we generated mutant 01310 PB2 genes with single amino acid mutations [PB2(01310)-I66M, PB2(01310)-K88R, PB2(01310)-I109V, PB2(01310)-I133V, PB2(01310)-R157K, PB2(01310)-K340R, PB2(01310)-L373I, PB2(01310)-V575M, PB2(01310)-E627K, and PB2(01310)-A674T] and tested their polymerase activity using an *in vitro* mini-genome assay in the 293T human embryonic kidney cell line (Fig. 1a). Among the tested mutations, only I133V and E627K significantly increased polymerase activity; these increases were 4- and 80-fold, respectively.

**Figure 2:**
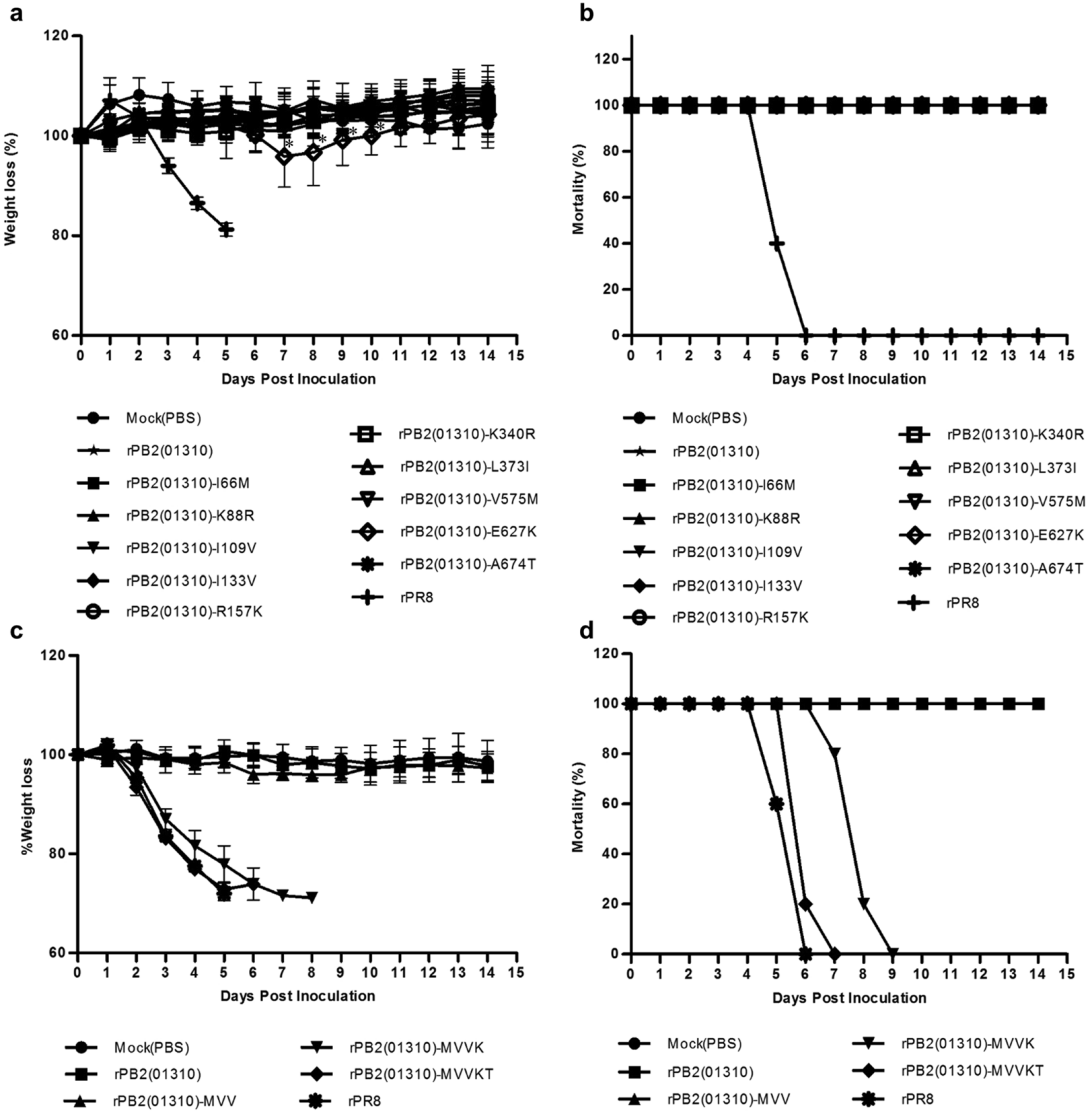
Comparison of mouse pathogenicity of 01310 PB2 variants. The virulence of 01310 PB2 single amino acid variants (**a**,**b**) and multi-amino acid variants (**c**,**d**) was observed by body weight loss (**a,c**) and mortality (**b,d**) of infected mice. Five 6-week-old BALB/c mice were challenged with 1.0 × 10^6^ EID_50_ of each virus or PBS (mock). Mortality and weight loss were observed for 14 days. The average weight loss ± s.d. was measured by comparing to the initial weight of each mouse. Statistical significance was analysed using Student’s t-test (compared to rPB2(01310), *P < 0.05).

To assess viral growth efficiency in MDCK cells and viral pathogenicity in mice, we generated PR8-derived recombinant viruses containing single amino acid mutations in 01310 PB2. Recombinant viruses with the I133V, L373I, or E627K mutations produced higher titres than the parent strain rPB2(01310) (*P* < 0.05) (Fig. 1b). In murine pathogenicity experiments, most of the mutant viruses, except the rPR8 virus, did not cause body weight loss or mortality during the observation period. Only rPB2(01310)-E627K caused slight body weight loss of up to 4% over 7-10 days post-infection (dpi) (*P* < 0.05) (Fig. 2a,b). During mouse infectivity screening, however, we observed detectable growth of mutant viruses containing the I66M, I109V, I133V, E627K, or A674T mutations (Table 1). The L373I mutation, which increases viral replication efficiency in MDCK cells, was excluded from further analysis because viruses carrying this mutation did not proliferate sufficiently in mouse lung. The E627K mutation has a well-characterized role related to mammalian pathogenicity, whereas the A674T mutation, which is conserved among most human influenza viruses, does not appear to be related to mammalian pathogenicity^23, 24^. 66M, 109V, and 133V are novel mutations that were first characterized in the present study.

**Table 1:** Comparison of the viral replication efficiencies of 01310 PB2 single amino acid variants in mouse lungs.

Virus	Positive rate* (Virus titre (logEID_50_/0.1 ml))		
	1^st^ experiment	2^nd^ experiment	
	3 dpi	3 dpi	6 dpi
Mock (PBS)	0/3(<0.5)	0/3(<0.5)	0/3(<0.5)
rPB2(01310)	0/3(<0.5)	0/3(<0.5)	0/3(<0.5)
rPB2(01310)-I66M	1/3(2.1)	0/3(<0.5)	0/3(<0.5)
rPB2(01310)-K88R	0/3(<0.5)	nt^†^	nt
rPB2(01310)-I109V	3/3(1.9)	2/3(1.7)	0/3(<0.5)
rPB2(01310)-I133V	3/3(2.5)	2/3(0.9)	3/3(3.5)
rPB2(01310)-R157K	0/3(<0.5)	nt	nt
rPB2(01310)-K340R	0/3(<0.5)	nt	nt
rPB2(01310)-L373I	1/3(<0.5)^‡^	nt	nt
rPB2(01310)-V575M	1/3(<0.5)	nt	nt
rPB2(01310)-E627K	3/3(4.1)	3/3(4.9)	3/3(5.3)
rPB2(01310)-A674T	1/3(1.7)	0/3(<0.5)	1/3(2.9)
rPR8	3/3(5.75)	3/3(6.1)	3/3(4.9)

*Number of positive samples/number of mice (virus titre of the pooled lung tissues, log_10_ EID_50_/0.1ml). ^†^Not tested. ^‡^Below the limit of assay detection.

### Frequency of key amino acid mutations

To understand the frequency of each identified mutation (I66M, I109V, I133V, E627K, and A674T), we examined IAVs collected from birds, pigs and humans (Table 2). The human IAVs were divided into two groups: the first contained the H5, H6, H7, H9, and H10 IAVs (birds to humans), and the second contained the H1, H2, and H3 IAVs (humans), excluding the 2009 pandemic H1N1 virus. Interestingly, most of the IAVs already possessed the 66M, 109V, or 133V mutation, regardless of the host species they were isolated from. However, viruses bearing the 627K and 674T mutations were significantly more common in ‘birds to humans’ and human IAVs than bird and pig IAVs. The well-known mammalian pathogenic factor, D701N mutation, occurs less frequently than the E627K mutation in the PB2 genes of “human” and “bird to human” IAVs (Table 2)^17, 25^. Thus, we concentrated on the effect of E627K and its synergistic effects with the MVV mutations with regard to mammalian pathogenicity.

**Table 2:** Frequency of residues in PB2 related to mammalian replication of influenza A virus between different hosts.

Host species	Frequency of amino acid residue (%)						Frequency of specific amino acid pattern at residues 66, 109, and 133 (%)						
	66M	109V	133V	627K	674T	701N	MVV*	MIV	IVV	MVI	MII	IVI	IIV
Birds (n = 12,561)	97.6^†^	95.4	99.6	2.8	0.7	0.1	92.7 (74.1^‡^/25.9^§^)	4.2 (86.0/14.0)	0.3 (39.5/60.5)	0.4 (3.3/96.7)	0.0	0.0	0.0
Pigs (n = 3,721)	87.4	92.1	99.9	8.0	1.7	15.9	85.8 (0.8/99.2)	1.2 (0/100)	4.6 (0/100)	0.0	0.0	0.0	0.0
Birds to humans (H5/H6/H7/H9/H10)(n = 299)	99.0	99.3	99.7	41.1	0.3	6.0	98.0 (16.7/83.3)	0.0	1.0 (0/100)	0.3 (0/100)	0.0	0.0	0.0
Humans (H1/H2/H3) (n = 9,161)	99.7	84.8	100.0	99.6	97.6	0.2	84.6 (0/100)	15.1 (0/100)	0.1 (0/100)	0.0	0.0	0.0	0.0

*Amino acid residues at positions 66, 109, and 133 in PB2. ^†^Percentage of viruses containing specific amino acid residues. ^‡^Percentage of viruses containing no mammalian pathogenicity-related mutations (9N, 147T, 158G, 192K, 199S, 253N, 271A, 339T, 404L, 526R, 588I/T, 590S, 591K/R, 627K, 674T, 701N, 702R, and/or 714R). ^§^Percentage of viruses containing mammalian pathogenicity-related mutations.

Among the combined novel amino acid mutations, the MVV group showed the highest frequencies in birds (92.7%), pigs (85.8%), and humans (birds to humans 98.0%; humans 84.6%). The MIV and IVV groups were the second most frequent in bird (4.2%), human (15.1%), pig (4.6%), and birds to humans (1.0%) IAVs. Furthermore, we calculated the ratios of viruses with or without additional amino acid substitutions related to the mammalian pathogenicity of AIV (9N, 147T, 158G, 192K, 199S, 253N, 271A, 339T, 404L, 526R, 588I/T, 590S, 591K/R, 627K, 674T, 701N, 702R, and 714R)^13, 24, 26–37^. The ratios of viruses with additional mammalian pathogenic factors were significantly lower in the MVV group of avian IAVs (25.9%) than in swine or human IAVs (pigs: 99.2%; birds to humans: 83.3%; humans: 100%) (*P* < 0.05). In contrast, the ratios of viruses without any additional mammalian pathogenic factors were significantly higher in the MVV group of avian IAVs (74.1%) than swine or human IAVs (pigs: 0.8%; birds to humans: 16.7%; humans: 0%) (*P* < 0.05).

### Effects of multiple amino acid mutations

Based on the high frequencies of the MVV and MIV mutations among the examined AIVs, we introduced multiple mutations combined with E627K and A674T into 01310 PB2 (MIV, MVV, MVVT, MIVKT, MVVK, and MVVKT) and performed mini-genome assays. The combination of 66M and 133V (MIV), as well as that of 66M, 109V, and 133V (MVV), significantly increased polymerase activity compared to the individual amino acid mutations at 33 °C and 37 °C (Fig. 1a and S1). The combination of MVV with 627K (MVVK) significantly increased polymerase activity compared to the single 627 K mutation at 33 °C and 37 °C (Fig. 1a and S1) (*P* < 0.05). The 674T mutation did not influence polymerase activity; however, in combination with MVV (MVVT), it increased polymerase activity significantly compared to MVV alone.

A panel of recombinant viruses was generated to assess viral replication efficiency in MDCK cells (Fig. 1b). Viruses containing the MIV [rPB2(01310)-MIV], MVV [rPB2(01310)-MVV], and MVVT [rPB2(01310)-MVVT] mutations showed significantly higher replication efficiency than rPB2(01310). The combinations of 627K or 627K and 674T with MIV (MIVKT) or MVV (MVVK and MVVKT) significantly increased the replication efficiencies of the corresponding recombinant viruses [rPB2(01310)-MIVKT, rPB2(01310)-MVVK, and rPB2(01310)-MVVKT] in MDCK cells compared to the 627K mutation alone [rPB2(01310)-E627K]. Furthermore, rPB2(01310)-MVV showed higher replication efficiency than rPB2(01310) in a porcine kidney cell line, PK-15, and a human lung adenocarcinoma cell line, A549; rPB2(01310)-MVVK showed higher replication efficiency than rPB2(01310)-MVV in PK-15 and A549 cells at 33 °C and 37 °C (Fig. S2).

In the murine pathogenicity experiments, rPB2(01310)-MVV successfully proliferated in lung tissue at 3 dpi (10^3.4^ EID_50_) and 6 dpi (10^3.0^ EID_50_), whereas rPB2(01310) did not (Table 3). Furthermore, compared to rPB2(01310)-E627K and rPB2(01310)-MVV, rPB2(01310)-MVVK and rPB2(01310)-MVVKT efficiently replicated in the lungs (10^5.8^ EID_50_ and 10^6.0^ EID_50_, respectively, at 3 dpi; 10^4.1^ EID_50_ and 10^4.4^ EID_50_, respectively, at 6 dpi) and caused severe body weight loss and 100% mortality (Table 3 and Fig. 2c,d). The mean time to death after rPB2(01310)-MVVKT inoculation was 6.2 ± 0.45 days, which was shorter than for rPB2(01310)-MVVK and similar to rPR8 virus (8 ± 0.71 days and 5.6 ± 0.55 days, respectively) (*P* < 0.05). Thus, the MIV and MVV mutations were demonstrated to have synergistic effects with 627K and 674T in the present study.

**Table 3:** Comparison of viral replication of PB2 variants in mouse lungs.

Virus	Mean titre (log EID_50_/0.1 ml*)	
	Lung	
	3 dpi	6 dpi
Mock (PBS)	<0.5	<0.5
rPB2(01310)	<0.5	<0.5
rPB2(01310)-MVV	3.4 ± 0.5	3.0 ± 1.5
rPB2(01310)-MVVK	5.8 ± 0.3	4.1 ± 0.4
rPB2(01310)-MVVKT	6.0 ± 0.4	4.4 ± 0.4
rPB2(PR8)-III	6.3 ± 0.5	5.0 ± 0.4
rPB2(PR8)-IIIET	1.4 ± 0.7	1.5 ± 0.7
rPR8	6.3 ± 0.2	4.5^†^
rPB2(PR8)-K627E	4.8 ± 0.7	4.6 ± 0.1

*Six BALB/c mice were inoculated with 10^6^ EID_50_ of each virus; three mice were euthanized at 3 and 6 days post inoculation. ^†^Data from only one mouse due to the death of two mice.

**Figure 3:**
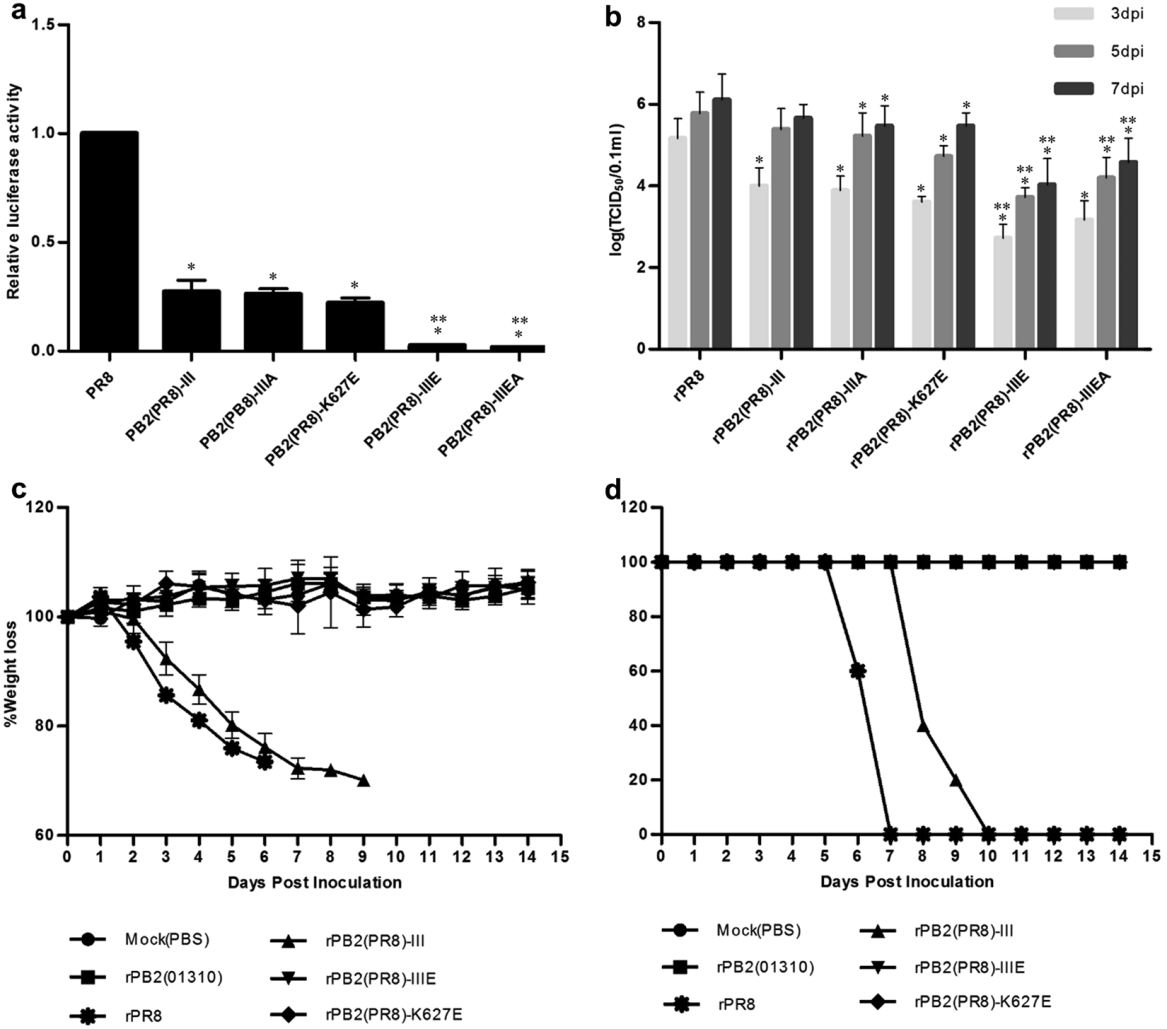
Effects of PR8 PB2 variants on mammalian adaptation. **(a**) Viral polymerase activity was measured using mini-genome assays in 293T cells. The data were normalized to the polymerase activity of wild-type PR8 PB2. The data presented are the average of three independent experiments ± s.d. from one experiment. Statistical significance was analysed using Student’s t-test (compared to the polymerase activity of PR8 PB2 gene, *P < 0.05; compared to the polymerase activity of PB2(PR8)-K627E, **P < 0.05). (**b**) Replication efficiency of PR8 PB2 variants in MDCK cells at 37 °C. Wild-type PR8 and mutant viruses were used to inoculate MDCK cells at 10^7^ EID_50_/0.1 ml, and the TCID_50_ was determined at 3, 5, and 7 dpi. The data presented are the average of three independent experiments ± s.d. from one experiment. Statistical significance was analysed by two-way analysis of variance with Bonferroni post-test correction (compared to PR8, *P < 0.05; compared to rPB2(01310)-K627E, **P < 0.05). The virulence of PR8 PB2 variants was observed based on the body weight loss (**c**) and mortality of infected mice (**d**). Five 6-week BALB/c mice were anaesthetized and challenged with 1.0 × 10^6^ EID_50_/50 $ul$ of each virus or PBS (mock). Mortality and weight loss were observed for 14 days. The average weight loss ± s.d. was measured by comparing to the initial weight of each mouse.

To further evaluate the effects of the MVV mutations, we constructed loss-of-function (LOF) genes using the mouse pathogenic PR8 PB2 gene (Fig. 3a). The polymerase activities of the LOF mutants possessing the 66I-109I-133I [PB2(PR8)-III], 66I-109I-133I-674A [PB2(PR8)-IIIA], or 627E [PB2(PR8)-K627E] mutations were significantly lower than that of the PR8 PB2 gene [PB2(PR8)]. Furthermore, the combination of III with 627E or 627E and 674A [PB2(PR8)-IIIE and PB2(PR8)-IIIEA] significantly decreased the polymerase activity compared to PB2(PR8)-III, PB2(PR8)-IIIA, and PB2(PR8)-K627E (*P* < 0.05).

We next generated LOF mutant viruses possessing the above-described mutated PR8 PB2 genes and compared their replication efficiency in MDCK cells. The virus titre of rPB2(PR8)-III at 3 dpi was slightly lower than that of rPR8, but it recovered at 5 and 7 dpi (Fig. 3b). Furthermore, rPB2(PR8)-IIIE and rPB2(PR8)-IIIEA replicated less efficiently than rPR8 and rPB2(PR8)-K627E.

Next, we assessed the pathogenicity of rPB2(PR8)-IIIE, rPB2(PR8)-III, and rPB2(PR8)-K627E in mice. rPB2(PR8)-III exhibited high virulence, similar to rPR8 (Fig. 3c,d). However, the mean time to death for rPB2(PR8)-III was 8.6 ± 0.89 days, which is approximately 2 days longer than that for rPR8 (6.6 ± 0.55 days) (*P* < 0.05). Moreover, the pathogenicity of rPB2(PR8)-IIIE was markedly attenuated; this variant caused no body weight loss and produced much lower virus titres in the lungs of infected mice than rPB2(PR8)-K627E and rPB2(PR8)-III (Table 3). These findings suggest that the MVV mutations in the PR8 PB2 protein have a substantial effect on viral polymerase activity and replication efficiency through synergistic cooperation with 627K.

The histopathological pulmonary lesions of mice infected with rPB2(01310), rPB2(01310)-E627K, rPB2(01310)-MVV, rPB2(01310)-MVVK, rPB2(PR8)-III, rPB2(PR8)-IIIE, and rPR8 were compared, and the average lesion scores of 5 mice per virus type, except rPB2(PR8)-III (4 mice due to 1 mouse death) and rPR8 (2 mice due to 3 mouse deaths), were calculated (Fig. S3). The pulmonary lesions in the rPB2(01310)-infected mice were similar to those in the mock group. Although the lesion score for rPB2(01310)-MVV did not significantly differ from that of rPB2(01310), rPB2(01310)-MVV induced mild to moderate pulmonary lesions in some infected mice. The pulmonary lesions induced by rPB2(01310)-E627K and rPB2(01310)-MVVK were characterized by necrotizing bronchiolitis, severe peribronchiolitis and interstitial pneumonia (average lesion scores 3.5 and 4, respectively), but rPB2(01310)-MVVK induced more severe necrotizing bronchiolitis in infected mice. rPR8 and rPB2(PR8)-III infection also caused necrotizing bronchiolitis, severe peribronchiolitis, interstitial pneumonia and bronchiolar epithelial proliferation (average lesion scores 3.5 and 4, respectively). These two viruses induced a similar degree of inflammation, but one rPR8-infected mouse showed more marked bronchiolar epithelial hyperplasia. The rPB2(PR8)-IIIE-infected mice showed less severe inflammation compared to the rPB2(PR8)-III- and rPR8-infected mice.

### Acquisition of fatal mammalian mutations

To investigate the effect of the MVV mutations on the acquisition of fatal mammalian adaptive mutations, we performed quasi-species analysis using the lungs of mice infected with rPB2(01310)-MVV at 6 dpi (Table S1). Cloned amplicons encoding amino acid residues 590-701 were sequenced, and well-known mammalian pathogenic mutations such as Q591R/K, E627K, and D701N were identified in four out of five lungs. Surprisingly, at least half of the quasi-species of three mice possessed mammalian pathogenic mutations (Q591K, E627K, or D701N), and double mutations [E627K (10/10) and Q591R (1/10)] were observed in one mouse.

### Host selectivity of mutant viruses

**Figure 4:**
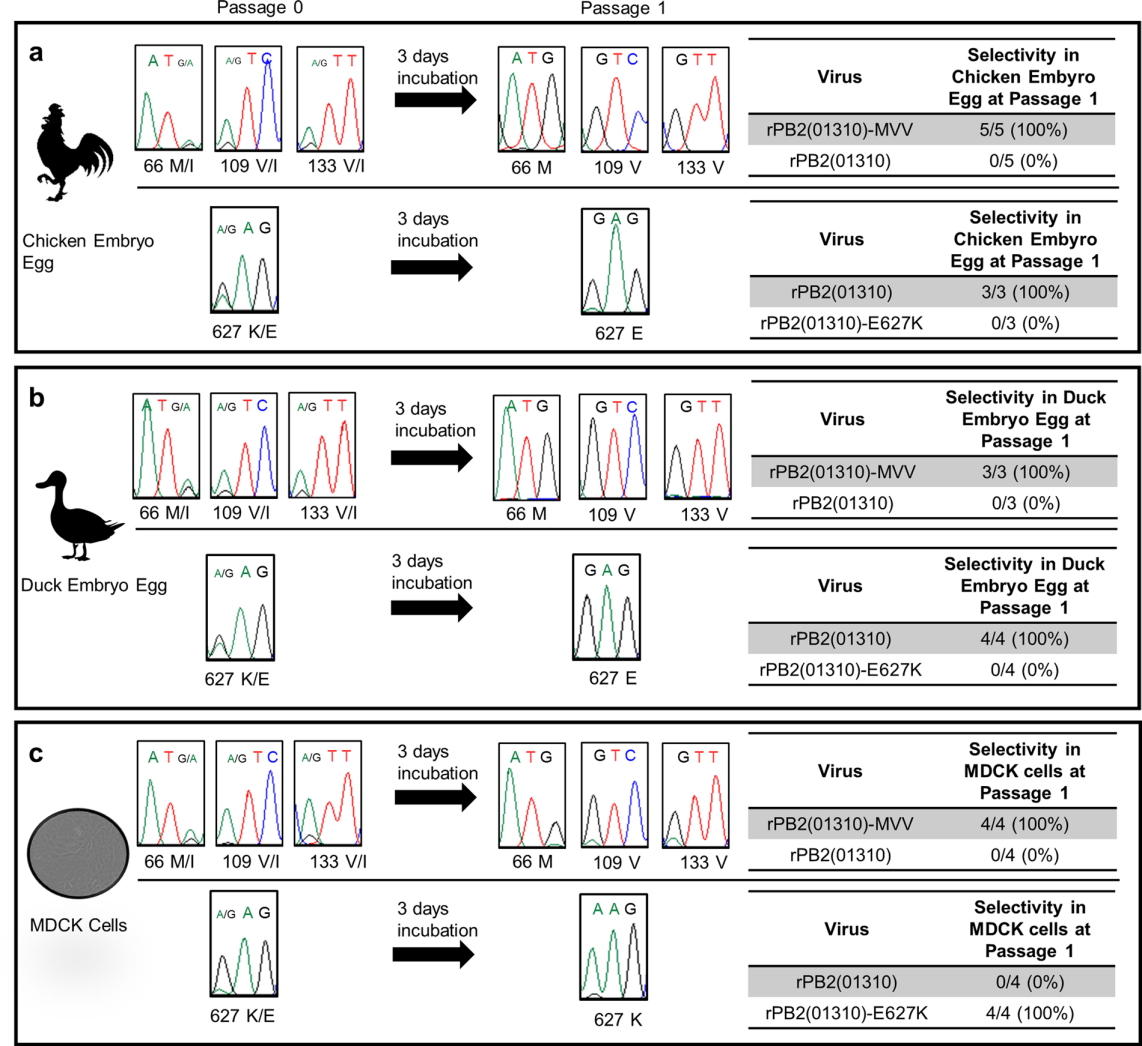
Comparison of the growth rate of each mutant virus against control virus in mixed cultures. Mixtures (1:1) of wild-type rPB2(01310) and rPB2(01310)-MVV (top) or rPB2(01310) and rPB2(01310)-E627K (bottom) were inoculated in ECEs (**a**), EDEs (**b**), and MDCK cells (**c**). At 3 dpi, the allantoic fluid of the embryonated eggs and the cell culture supernatants of the MDCK cells were collected, and the PB2 gene was amplified by RT-PCR and sequenced. The codon for 66I is ATA, 109I is ATC, 133I is ATT, and 627E is GAG; for the PB2 variants, the codon for 66M is ATG, 109V is GTC, 133V is GTT, and 627K is AAG. The DNA sequence chromatograms shown correspond to one sample from each host.

To compare the relative replication efficiencies of rPB2(01310) and rPB2(01310)-MVV in mammalian and avian hosts, we mixed equal titres of both viruses and inoculated them into cultured MDCK cells, 10-day-old embryonated chicken eggs (ECEs), and embryonated duck eggs (EDEs). EDEs were used in addition to ECEs to better generalize our results among different avian hosts. Culture media supernatant and allantoic fluid were collected from the ECEs and EDEs and subjected to sequencing analysis, along with the pre-inoculation virus mixture. According to four or five independent experiments, rPB2(01310)-MVV outgrew rPB2(01310) in both mammalian (MDCK cells, 4/4) and avian (ECEs and EDEs, 5/5) hosts after only one passage (Fig. 4). In the same experimental context, we mixed equal titres of rPB2(01310) and rPB2(01310)-E627K and inoculated MDCK cells, ECEs, and EDEs. rPB2(01310)-E627K outgrew rPB2(01310) in MDCK cells (4/4), but rPB2(01310) outgrew rPB2(01310)-E627K in ECEs and EDEs (5/5) after only one passage (Fig. 4).

### Structure-function relationship

**Figure 5:**
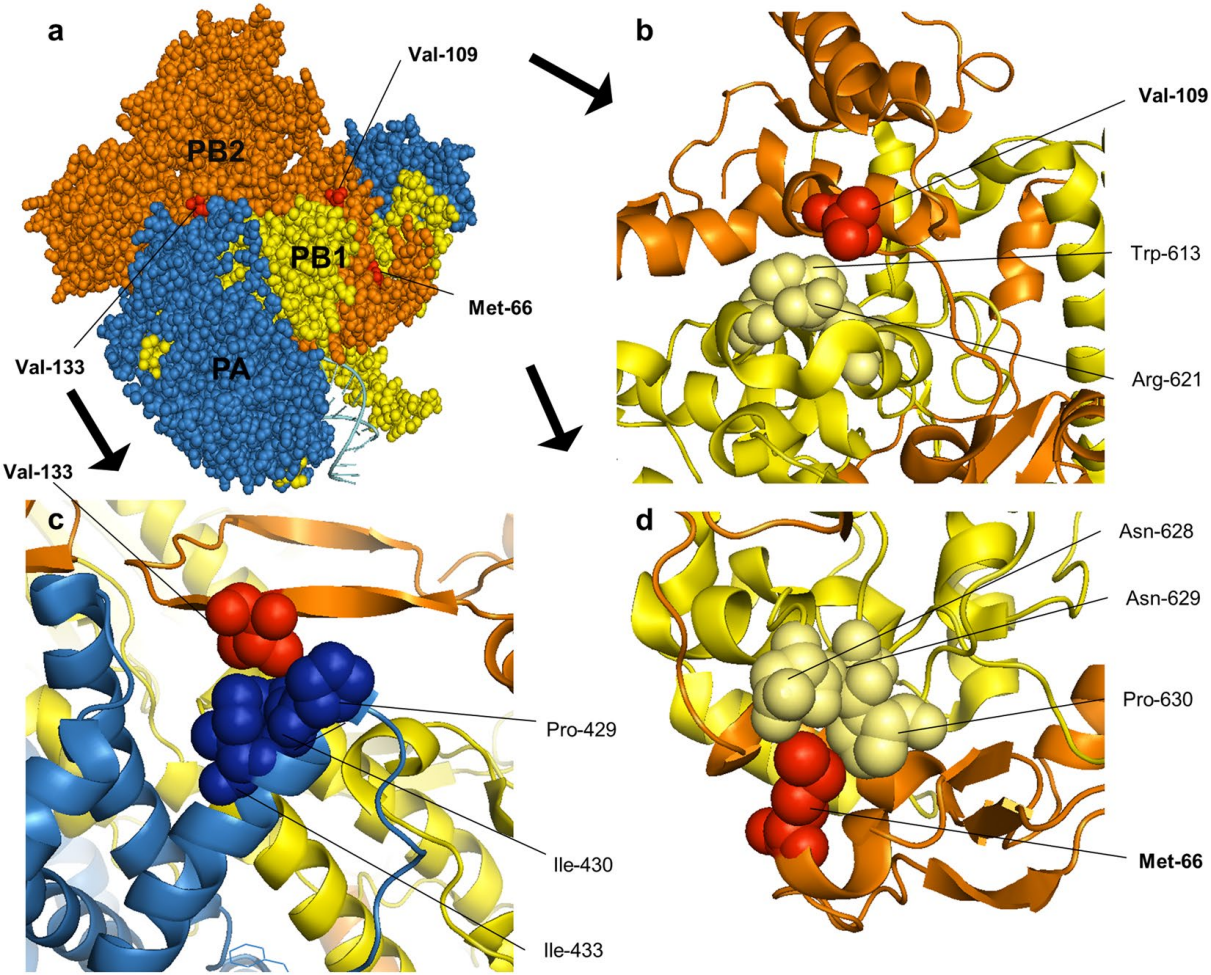
Locations of residues 66, 109, and 133 near the PB1-PB2 and PA-PB2 interfaces in the polymerase complex model. The crystal structure of the A/little yellow-shoulder bat/Guatemala/060/2010 (H17N10) polymerase complex (PDB code: 4WSB) was used to locate amino acid residues in IAV. The analysis was performed using the program PYMOL (W. L. Delane; http://www.pymol.org). (**a**) The locations of the 66,109, and 133 residues in the polymerase complex. The interface of PB1-PB2 with the residues 109V (**b**) and 66M (**d**). The interface of PA-PB2 with the 133V (**c**) residue. Each subunit of the polymerase complex is shown in a different colour.

To predict the structure-function relationship of I66M, I109V, and I133V, we located each amino acid residue in a 3D structure of the PB1, PB2, and PA trimer from a bat IAV^3^. Interestingly, all the residues at the 66th, 109th, and 133rd positions were predicted to be located at the interfaces of the polymerase complex (Fig. 5). The 66th and 109th residues were located close to 628N, 629N, and 630P as well as 613W and 621R of the PB1 protein. The 133rd residue was located close to 429P, 430I, and 433I of PA. We compared the neighbouring amino acid residues of bat IAV PB1 and PA with those of 01310 and PR8 PB1 and PA and found that most (except 621 and 628 of PB1) were conserved. Residues 621 and 628 of 01310 and PR8 are glutamine and leucine, respectively. By analysing the frequencies of the neighbouring amino acid residues in PB1 and PA, it was demonstrated that the 613W, 629N, 628L/M, and 630P residues of most IAVs are located in PB1 regardless of host species, but position 621 in PB1 carries different amino acids depending on the host species (Table S2). Residues 429P, 430I, and 433I in PA were conserved among most avian and mammalian IAVs (434P, 435I, and 438I), but the precise locations differed due to a 5-amino-acid deletion in bat PA. Considering that the neighbouring amino acids are conserved, except for residue 621 in PB1, these residues may form key interactions with 66M, 109V, and 133V. In other words, the I66M, I109V, and I133V mutations may affect the structural integrity of the polymerase complex.

## Discussion

To date, various amino acid changes in avian IAVs related to mammalian replication and pathogenicity have been reported, and PB2 is one of the most important genes for overcoming host barrier defences^36, 38^. In the present study, we identified the novel amino acid mutations I66M, I109V, and I133V, which increased polymerase activity and replication efficiency in mammalian and avian hosts as well as pathogenicity in mice both independently and in combination. The E627K mutation was previously reported to play a key role in the mammalian pathogenicity of avian IAVs^10, 14, 15, 37^. The single E627K mutation in the prototypic 01310 PB2 increased polymerase activity and replication efficiency more effectively than the MVV mutations, but it did not cause apparent body weight loss or mortality in mice (Figs 1a,b and 2a,b). However, the combination of E627K with the MVV mutations was sufficient to cause severe body weight loss and mortality (Fig. 2c,d). Although the MDT of the LOF mutant rPB2(PR8)-III was prolonged, the mutant retained sufficient pathogenicity to cause 100% mortality in mice. However, the K627E mutation eliminated the pathogenicity of the LOF mutants rPB2(PR8)-K627E and rPB2(PR8)-IIIE in mice (Fig. 3c,d). Thus, the E627K mutation has more important implications than the MVV mutations, but the MVV mutations are present at a much higher frequency than E627K in avian, swine, and human IAVs and therefore may represent the first-step mutations acquired by the prototypic PB2. Based on the growth competition between MVV and III recombinant viruses, the MVV mutations may be required for efficient replication in avian as well as mammalian hosts. Therefore, the very low frequencies of the intermediate mutants MIV, MVI, IVV, MII, IVI, and IIV and the prototype III in IAVs may imply that the evolution of polymerase activity in birds is approaching ‘evolutionary stasis’, raising a question about the presence of other hosts of ancient IAVs and their transmission to modern birds and mammals (Table 2)^2^.

The significantly lower frequencies of MVV and MIV with additional mammalian pathogenicity mutations in birds compared to pigs and humans may reflect negative selection in birds, but the significantly higher frequencies of MVV and MIV with additional mammalian pathogenicity mutations in pigs and humans may reflect positive selection (Table 2). The different selectivity of the 627E and 627K mutations in avian and mammalian hosts, respectively, has been a matter of conflict^39–44^. In addition, the 627K mutation increased the mammalian pathogenicity of most H5N1 IAVs, but it did not increase the pathogenicity of other IAVs, including equine and some swine IAVs, some H5N1 viruses, and pandemic H1N1 viruses^10, 45–47^. The growth competition experiment clearly demonstrated that viruses harbouring 627E grew more rapidly than those harbouring 627K in avian hosts and that those harbouring 627K grew more rapidly than those harbouring 627E in mammalian cells (Fig. 4). Thus, the conflicting results of previous reports may be caused by different backgrounds of additional mammalian pathogenicity mutations^48^. Therefore, the prototypic 01310 PB2 may be useful for comparing the effects of mammalian pathogenicity mutations on viral growth efficiency in different hosts and pathogenicity in mammals.

Our quasi-species study revealed that the MVV mutations were the minimum requirement for the acquisition of additional mammalian pathogenicity mutations. Acquisition of additional E627K, D701N, or Q591K/R mutations in 4 out of 5 mice during the first mammalian infection was unexpected, but it also supports the fact that the MVV mutations are the minimum essential predisposing mutations to acquire mutations for mammalian pathogenicity. When we compared the mammalian pathogenicity mutation patterns in PB2 from pig and human IAVs, it was found that multiple mutations in different combinations had been accumulated in PB2 (data not shown). These different accumulation patterns may be the result of growth competition in natural hosts. Therefore, tracking of accumulated mutations in PB2 using the prototypic 01310 PB2 may provide information on the effect of single or accumulated mutations on mammalian pathogenicity, providing some insight into the evolutionary steps taken by PB2 during adaptation to mammals under competition between IAVs.

According to computational structure-function analysis, the 66th, 109th, and 133rd residues were predicted to be located at the interfaces of the polymerase complex of a bat IAV (Fig. 5)^3^. The conserved amino acids in PB1 and PA neighbouring the residues at positions 66, 109, and 133 of PB2 supports the importance of the I66M, I109V, and I133V mutations. The position 66 isoleucine and methionine have hydrophobic side chains, but methionine may interact more strongly with neighbouring 628L and 630P. Because the side chain of isoleucine is bulkier than that of valine, 109V and 133V may fit better into the interface of PB1 and PA, which may affect the structural integrity of the polymerase complex and thereby increase polymerase activity. Therefore, the MVV mutations are not host-specific but rather universal mutations for avian and mammalian host adaptation. The question of why the polymerase evolved to obtain greater structural integrity remains to be answered. However, a recent report indicated that oviraptorids, ancient relatives of birds in the Cretaceous period, had a lower body temperature than modern birds and mammals, which could have necessitated a more structurally stable complex^49^.

In conclusion, the MVV mutations in PB2 have an important role in IAV replication. These mutations not only affect the structural integrity of the polymerase complex but are also an essential prerequisite for the subsequent acquisition of mammalian pathogenic mutations. Our results raise questions surrounding how the prototypic PB2 was transmitted from ancient hosts, with lower body temperatures, to modern birds, with higher body temperature. In addition to improving understanding of the molecular steps taken by IAV to acquire mammalian pathogenicity, the prototypic PB2 may be useful as a template for the grafting of certain mutations to compare their effects on mammalian pathogenicity.

## Materials and Methods

### Viruses, eggs, and cells

This study used A/PR/8/34 (H1N1) (PR8) virus and A/chicken/Korea/01310/2001 (H9N2) (01310), a strain used for an inactivated oil emulsion vaccine in Korea. To synthesize recombinant PR8 virus (rPR8), a Hoffmann vector system was used as described previously^50, 51^. Recombinant PR8 viruses were generated and passaged three times in 10-day-old SPF embryonated chicken eggs (ECEs) (Charles River Laboratories, North Franklin, USA) and then used in experiments. Additionally, 293T, MDCK, and A549 cells were purchased from the Korean Collection for Type Cultures (KCTC, Daejeon, Korea), and PK-15 cell was acquired from American Type Culture Collection (ATCC, VA, USA). 293T, MDCK, and PK-15 cells were maintained in DMEM supplemented with 10% FBS (Life Technologies Co., CA, USA), and A549 cell was maintained in DMEM/F12 supplemented with 10% FBS.

### Cloning of the PB2 gene and site-directed mutagenesis of 01310 PB2 genes

Each viral segment was cloned into Hoffmann’s bi-directional transcription vector pHW2000^50^. The insert sequence was confirmed by sequencing with primers cmv-SF (5′-TAAGCAGAGCTCTCTGGCTA-3′) and bGH-SR (5′-TGGTGGCGTTTTTGGGGACA-3′). Site-directed mutagenesis of specific amino acid substitutions in PB2 genes from the 01310 virus was implemented using a Muta-direct Site Directed Mutagenesis Kit (iNtRON, Korea) as per the manufacturer’s protocol.

### Mini-genome assay

To evaluate the polymerase activity of each mutated virus, we constructed pHW-NP-Luc plasmids, which have pHW2000 backbones containing the untranslated region of the PR8 NP gene inserted in the antisense direction between the RNA polymerase I promoter and the terminator of the vector. The firefly luciferase gene from the pGL-3 vector was inserted between the NP gene 5′ and 3′ non-coding regions. 293T cells in 12-well plates were co-transfected with 0.1 *μ*g each of pHW-NP-Luc and mutated 01310 PB2 and PR8 PB1, PA and NP genes. Additionally, 0.1 *μ*g of the *Renilla* luciferase plasmid pRL-TK (Promega, USA) was also co-transfected, which served as an internal control to normalize variations in transfection efficiency and sample processing. Then, 24 hours after transfection, luminescence was assessed using a Dual-Glo Luciferase Assay System (Promega, USA) in accordance with the manufacturer’s instructions on a TECAN Infinite200 pro machine (Tecan Benelux bv, Giessen, Netherlands). All results shown are the average from triplicate experiments, and the standard deviation was calculated.

### Rescue of mutant viruses

PB2 plasmids containing mutations of interesting amino acids and 7 genome segments of PR8 were transfected into 293T cells by transfecting Hoffmann’s eight reverse genetics plasmids as described previously with some modifications^50, 51^. Briefly, bi-directional pHW2000 vectors containing PB1, PA, HA, NP, M, and NS of the PR8 virus and 01310 virus PB2 containing mutations in amino acids of interest were transfected with 300 ng of each plasmid using Lipofectamine 2000 and Plus reagents (Life Technologies Co., CA, USA). After overnight incubation, 1 ml of Opti-MEM (Life Technologies Co., CA, USA) and 0.5 mg/ml of L-1-tosylamido-2-phenylethyl chloromethyl ketone (TPCK)-treated trypsin (Sigma-Aldrich, USA) were added. After 24 h, the culture medium was harvested, and 200 µl of the medium was injected into 10-day-old SPF ECEs via the allantoic cavity. Three days after inoculation, the allantoic fluid was harvested and checked for virus growth via HA assay using 1% (v/v) chicken red blood cells (RBCs) according to the WHO Manual on Animal Influenza Diagnosis and Surveillance. All mutant viruses were confirmed by RT-PCR and sequencing.

### Titration of viruses

Each mutant virus was inoculated into five 10-day-old SPF ECEs for virus isolation. To estimate virus titre, each virus was serially diluted from 10^−1^ to 10^−9^ in 10-fold increments, and each dilution was injected into five 10-day-old SPF ECEs as well as inoculated onto MDCK cells. The 50% chicken embryo infectious dose (EID_50_) and 50% tissue culture infectious dose (TCID_50_) were calculated using the Spearman-Karber method.

### Comparative replication efficiency in mammalian cells

To evaluate the replication efficiency of each virus, MDCK (2 × 10^4^/ml), PK-15 (2.5 × 10^4^/ml), and A549 (2 × 10^4^/ml) cells were seeded in 96-well plates (100 $ul$/well). After 24 hours, confluent cells were washed twice with phosphate-buffered saline (PBS). Mutant viruses at 10^7^ EID_50_/0.1 ml were serially diluted from 10^−1^ to 10^−8^ in 10-fold increments, and 200 $ul$ of each dilution was inoculated into each well with DMEM supplemented with 1% bovine serum albumin (BSA) (fraction V) (Roche, Basel, Switzerland), 20 mM HEPES, antibiotic-antimycotic (Gibco, CA, USA), and 1 µg/ml (for MDCK cells), 0.5 µg/ml (for PK-15 cells), and 0.25 µg/ml (for A549 cells) TPCK-treated trypsin (Sigma-Aldrich, USA). The supernatants of the virus-infected cells were collected at 3, 5 and/or 7 dpi, and virus growth efficiency was determined by calculating the TCID_50_ using the Spearman-Karber method. Values are presented as the average of three independent experiments ± s.d.

### Animal experiments

Six-week-old female BALB/c mice were purchased from KOATEC (Pyeongtaek, Korea), and a mouse pathogenicity test was carried out by BIoPOA Co (Yongin, Korea) in accordance with national guidelines for the care and use of laboratory animals. To measure the mouse pathogenicity of each mutant virus, five mice were anaesthetized via intraperitoneal injection of 15 mg/kg Zoletil 50 (Virbac, Carros, France) and then intranasally inoculated with 10^6^ EID_50_/50 µl of each virus as described previously^22^. Negative control (mock) mice were injected with the same volume of sterilized PBS. Mortality and weight loss were measured for 14 days. Mice that lost more than 30% of their original weight were euthanized and recorded as a death. For the measurement of virus replication in the lungs of infected mice, six mice from each group were injected with PBS (mock) or 10^6^ EID_50_/50 µl of mutant virus. The lungs were collected at 3 and 6 dpi and then stored at −70 °C until use. The lungs were ground using a TissueLyzer 2 (Qiagen, Valencia, CA, USA) with 5 mm stainless steel beads and a volume of PBS equal to 10% of the lung weight in suspension. Then, 10 volumes of PBS were mixed with the ground tissues. After centrifugation at 2000 × g for 10 min, the supernatants were used for viral titres, which were measured as described above. To determine whether adaptive mutations were present in the PB2 gene of viruses isolated from infected mouse lung, RNA extraction and RT-PCR were carried out using lung diluents infected with rPB2(01310)-MVV. The PCR products were cloned into a TA cloning vector (RBC, Taiwan). By selecting 10 colonies per specimen and sequencing, the proportions of adaptive mutations were confirmed.

### Histopathology

Mice from each group were euthanized on day 6 dpi, and lungs were collected and fixed in 10% phosphate-buffered neutral formalin. One slice from each lung lobe per mouse was pulled, processed routinely, embedded in paraffin, and stained with haematoxylin and eosin for histopathological analyses. Histological changes were evaluated according to the modified methods of McAuley *et al*.^52^. Each pulmonary lesion was graded on a scale of 0 to 4. The grading system for histological characterization of the lesions was defined as follows: 0, normal lung; 1, mild infiltration of inflammatory cells around airways and vessels; 2, moderate infiltration of inflammatory cells around airways and vessels and mild leukocyte infiltration of alveolar spaces and interstitium; 3, moderate to severe infiltration of inflammatory cells around airways and vessels, moderate leukocyte infiltration of alveolar spaces and interstitium, mild necrosis, and hyperplasia of airway epithelium; 4, severe infiltration of inflammatory cells around airways and vessels, severe leukocyte infiltration of alveolar spaces and interstitium, moderate to severe necrosis, and hyperplasia of airway epithelium.

### Frequencies of specific amino acids in avian, human, and swine viruses

To evaluate the frequencies of specific amino acids from avian and mammalian hosts, full-length bird (PB2, n = 12,561; PB1, n = 13,614; PA, n = 13,921), pig (PB2, n = 3,721; PB1, n = 4,047; PA, n = 4,164), and human (PB2, n = 9,460; PB1, n = 17,423; PA, n = 17,639) IAV ORF sequences corresponding to the viral polymerase genes were acquired from the NCBI Influenza Virus Resource (http://www.fludb.org). We analysed the PB2, PB1, and PA sequences of several host influenza viruses using complete genome sequences and excluded all pandemic H1N1 sequences in order to eliminate bias. Human influenza viruses were divided into two groups: one group contained the H1, H2 and H3 subtypes (PB2, n = 9,161; PB1, n = 17,134; PA, n = 17,335), and the other group contained the H5, H6, H7, H9, and H10 subtypes (PB2, n = 299; PB1, n = 289; PA, n = 304). Sequences were aligned using CLUSTALW, and amino acid frequencies were compared between avian and mammalian influenza viruses.

### Competitive replicative ability of mutant viruses in embryonated chicken and duck eggs and in MDCK cells

To confirm the host suitability of each mutant virus, 100 EID_50_ of mutant virus and control virus were mixed and inoculated into 10-day-old SPF ECEs, 14-day-old EDEs, and MDCK cells were inoculated with virus at an MOI of 1. At 3 dpi, allantoic fluid or cell culture supernatant were harvested and sequenced. By comparing the peaks of the DNA sequence chromatograms, the most abundant codons were estimated.

### Molecular modelling

The crystal structure of the A/little yellow-shouldered bat/Guatemala/060/2010(H17N10) polymerase complex (PDB code: 4WSB) was used to locate amino acid residues in the IAV polymerase model with the PyMOL Molecular Graphics System (Version 1.1, DeLano Scientific LLC).

### Ethics statement

All mouse experiments were carried out at BioPOA Co. (Yongin, Korea) following a protocol that adhered to the National Institutes of Health’s Public Health Service Policy on the Humane Care and Use of Laboratory Animals. The protocol was reviewed and approved by the Institutional Animal Care and Use Committee (IACUC) of BioPOA Co. (BP-2014-0006-2, BP-2016-006-2).

## Electronic supplementary material

Supplementary information

## References

[1] Fouchier RA (2005). Characterization of a novel influenza A virus hemagglutinin subtype (H16) obtained from black-headed gulls. Journal of virology.

[2] Webster RG, Bean WJ, Gorman OT, Chambers TM, Kawaoka Y (1992). Evolution and ecology of influenza A viruses. Microbiol Rev.

[3] Pflug A, Guilligay D, Reich S, Cusack S (2014). Structure of influenza A polymerase bound to the viral RNA promoter. Nature.

[4] Tong S (2013). New world bats harbor diverse influenza A viruses. PLoS Pathog.

[5] Claas EC (1998). Human influenza A H5N1 virus related to a highly pathogenic avian influenza virus. Lancet.

[6] Detjen BMSt, Angelo C, Katze MG, Krug RM (1987). The three influenza virus polymerase (P) proteins not associated with viral nucleocapsids in the infected cell are in the form of a complex. Journal of virology.

[7] Gonzalez S, Zurcher T, Ortin J (1996). Identification of two separate domains in the influenza virus PB1 protein involved in the interaction with the PB2 and PA subunits: a model for the viral RNA polymerase structure. Nucleic acids research.

[8] Guilligay D (2008). The structural basis for cap binding by influenza virus polymerase subunit PB2. Nat Struct Mol Biol.

[9] Poch O, Sauvaget I, Delarue M, Tordo N (1989). Identification of four conserved motifs among the RNA-dependent polymerase encoding elements. The EMBO journal.

[10] Hatta M, Gao P, Halfmann P, Kawaoka Y (2001). Molecular basis for high virulence of Hong Kong H5N1 influenza A viruses. Science.

[11] Massin P, van der Werf S, Naffakh N (2001). Residue 627 of PB2 is a determinant of cold sensitivity in RNA replication of avian influenza viruses. J Virol.

[12] Snyder MH, Buckler-White AJ, London WT, Tierney EL, Murphy BR (1987). The avian influenza virus nucleoprotein gene and a specific constellation of avian and human virus polymerase genes each specify attenuation of avian-human influenza A/Pintail/79 reassortant viruses for monkeys. Journal of virology.

[13] Subbarao EK, London W, Murphy BR (1993). A single amino acid in the PB2 gene of influenza A virus is a determinant of host range. Journal of virology.

[14] Zhang H (2014). The PB2 E627K mutation contributes to the high polymerase activity and enhanced replication of H7N9 influenza virus. J Gen Virol.

[15] Kanta Subbarao, E., Brian, W. L. & Murphy, R. A single amino acid in the PB2 gene of influenza A virus is a determinant of host range. *Journal of Virology* 1764–1764 (1993). 10.1128/jvi.67.4.1761-1764.1993 PMC240216 8445709

[16] Kuzuhara T (2009). Structural basis of the influenza A virus RNA polymerase PB2 RNA-binding domain containing the pathogenicity-determinant lysine 627 residue. J Biol Chem.

[17] Steel J, Lowen AC, Mubareka S, Palese P (2009). Transmission of influenza virus in a mammalian host is increased by PB2 amino acids 627K or 627E/701N. PLoS Pathog.

[18] Tarendeau F (2008). Host determinant residue lysine 627 lies on the surface of a discrete, folded domain of influenza virus polymerase PB2 subunit. PLoS Pathog.

[19] Gabriel G (2011). Differential use of importin-alpha isoforms governs cell tropism and host adaptation of influenza virus. Nature communications.

[20] Labadie K, Dos Santos Afonso E, Rameix-Welti MA, van der Werf S, Naffakh N (2007). Host-range determinants on the PB2 protein of influenza A viruses control the interaction between the viral polymerase and nucleoprotein in human cells. Virology.

[21] Rameix-Welti MA, Tomoiu A, Dos Santos Afonso E, van der Werf S, Naffakh N (2009). Avian Influenza A virus polymerase association with nucleoprotein, but not polymerase assembly, is impaired in human cells during the course of infection. Journal of virology.

[22] Kim IH, Choi JG, Lee YJ, Kwon HJ, Kim JH (2014). Effects of different polymerases of avian influenza viruses on the growth and pathogenicity of A/Puerto Rico/8/1934 (H1N1)-derived reassorted viruses. Vet Microbiol.

[23] Miotto O, Heiny A, Tan TW, August JT, Brusic V (2008). Identification of human-to-human transmissibility factors in PB2 proteins of influenza A by large-scale mutual information analysis. BMC Bioinformatics.

[24] Bussey KA, Bousse TL, Desmet EA, Kim B, Takimoto T (2010). PB2 residue 271 plays a key role in enhanced polymerase activity of influenza A viruses in mammalian host cells. J Virol.

[25] Zhu W (2015). Dual E627K and D701N mutations in the PB2 protein of A(H7N9) influenza virus increased its virulence in mammalian models. Sci Rep.

[26] Gabriel G (2005). The viral polymerase mediates adaptation of an avian influenza virus to a mammalian host. Proc Natl Acad Sci USA.

[27] Li Z (2005). Molecular basis of replication of duck H5N1 influenza viruses in a mammalian mouse model. J Virol.

[28] Yamada S (2010). Biological and structural characterization of a host-adapting amino acid in influenza virus. PLoS Pathog.

[29] Mehle A, Doudna JA (2009). Adaptive strategies of the influenza virus polymerase for replication in humans. Proc Natl Acad Sci USA.

[30] Zhao Z (2014). PB2-588I enhances 2009 H1N1 pandemic influenza virus virulence by increasing viral replication and exacerbating PB2 inhibition of beta interferon expression. J Virol.

[31] Song W (2014). The K526R substitution in viral protein PB2 enhances the effects of E627K on influenza virus replication. Nat Commun.

[32] Liu Q (2015). Virulence determinants in the PB2 gene of a mouse-adapted H9N2 virus. J Virol.

[33] Mok CK (2011). Amino acid residues 253 and 591 of the PB2 protein of avian influenza virus A H9N2 contribute to mammalian pathogenesis. J Virol.

[34] Taft AS (2015). Identification of mammalian-adapting mutations in the polymerase complex of an avian H5N1 influenza virus. Nat Commun.

[35] Zhou B (2011). PB2 residue 158 is a pathogenic determinant of pandemic H1N1 and H5 influenza a viruses in mice. J Virol.

[36] Fan S (2014). Novel residues in avian influenza virus PB2 protein affect virulence in mammalian hosts. Nat Commun.

[37] Kim JH (2010). Role of host-specific amino acids in the pathogenicity of avian H5N1 influenza viruses in mice. J Gen Virol.

[38] Schrauwen EJ, Fouchier RA (2014). Host adaptation and transmission of influenza A viruses in mammals. Emerg Microbes Infect.

[39] Shinya K (2004). PB2 amino acid at position 627 affects replicative efficiency, but not cell tropism, of Hong Kong H5N1 influenza A viruses in mice. Virology.

[40] Long JS (2013). The effect of the PB2 mutation 627K on highly pathogenic H5N1 avian influenza virus is dependent on the virus lineage. Journal of virology.

[41] Bortz, E. *et al*. Host- and strain-specific regulation of influenza virus polymerase activity by interacting cellular proteins. *MBio* **2**, doi:10.1128/mBio.00151-11 (2011). 10.1128/mBio.00151-11 PMC3157893 21846828

[42] Chin AW (2014). Influenza A viruses with different amino acid residues at PB2-627 display distinct replication properties *in vitro* and *in vivo*: revealing the sequence plasticity of PB2-627 position. Virology.

[43] Hudjetz B, Gabriel G (2012). Human-like PB2 627K influenza virus polymerase activity is regulated by importin-alpha1 and -alpha7. PLoS Pathog.

[44] Mok CK (2014). Amino acid substitutions in polymerase basic protein 2 gene contribute to the pathogenicity of the novel A/H7N9 influenza virus in mammalian hosts. J Virol.

[45] Gao P (1999). Biological heterogeneity, including systemic replication in mice, of H5N1 influenza A virus isolates from humans in Hong Kong. Journal of virology.

[46] Schnitzler SU, Schnitzler P (2009). An update on swine-origin influenza virus A/H1N1: a review. Virus Genes.

[47] Shinya K, Watanabe S, Ito T, Kasai N, Kawaoka Y (2007). Adaptation of an H7N7 equine influenza A virus in mice. J Gen Virol.

[48] Foeglein A (2011). Influence of PB2 host-range determinants on the intranuclear mobility of the influenza A virus polymerase. The Journal of general virology.

[49] Eagle RA (2015). Isotopic ordering in eggshells reflects body temperatures and suggests differing thermophysiology in two Cretaceous dinosaurs. Nature communications.

[50] Hoffmann E, Neumann G, Kawaoka Y, Hobom G, Webster RG (2000). A DNA transfection system for generation of influenza A virus from eight plasmids. Proc Natl Acad Sci USA.

[51] Hoffmann E, Krauss S, Perez D, Webby R, Webster RG (2002). Eight-plasmid system for rapid generation of influenza virus vaccines. Vaccine.

[52] McAuley JL (2007). Expression of the 1918 influenza A virus PB1-F2 enhances the pathogenesis of viral and secondary bacterial pneumonia. Cell Host Microbe.

