# Supplementary material for: Prerequisites for the acquisition of mammalian pathogenicity by influenza A virus with a prototypic avian PB2 gene
Source: Sci Rep. 2017 Aug 31;7:10205. doi: 10.1038/s41598-017-09560-z (PMC5579056; doi:10.1038/s41598-017-09560-z)
Supplement: Supplementary file 1 — Supplementary information [file 41598_2017_9560_MOESM1_ESM.doc]

**Supplementary information**

**Prerequisites for the acquisition of mammalian pathogenicity by influenza A virus with a prototypic avian PB2 gene**

**Authors:** Chung-Young Lee, Se-Hee An, Ilhwan Kim, Du-Min Go, Dae-Yong Kim, Jun-Gu Choi, Youn-Jeong Lee, Jae-Hong Kim, Hyuk-Joon Kwon

Figure S1 Page 2

Figure S2 Page 3

Figure S3 Page 4-5

Table S1 Page 6

Table S2 Page 7

**Supplementary Figure and Figure legend**

**Figure S1. Viral polymerase activity of 01310 PB2 variants at 33 ℃.**

**
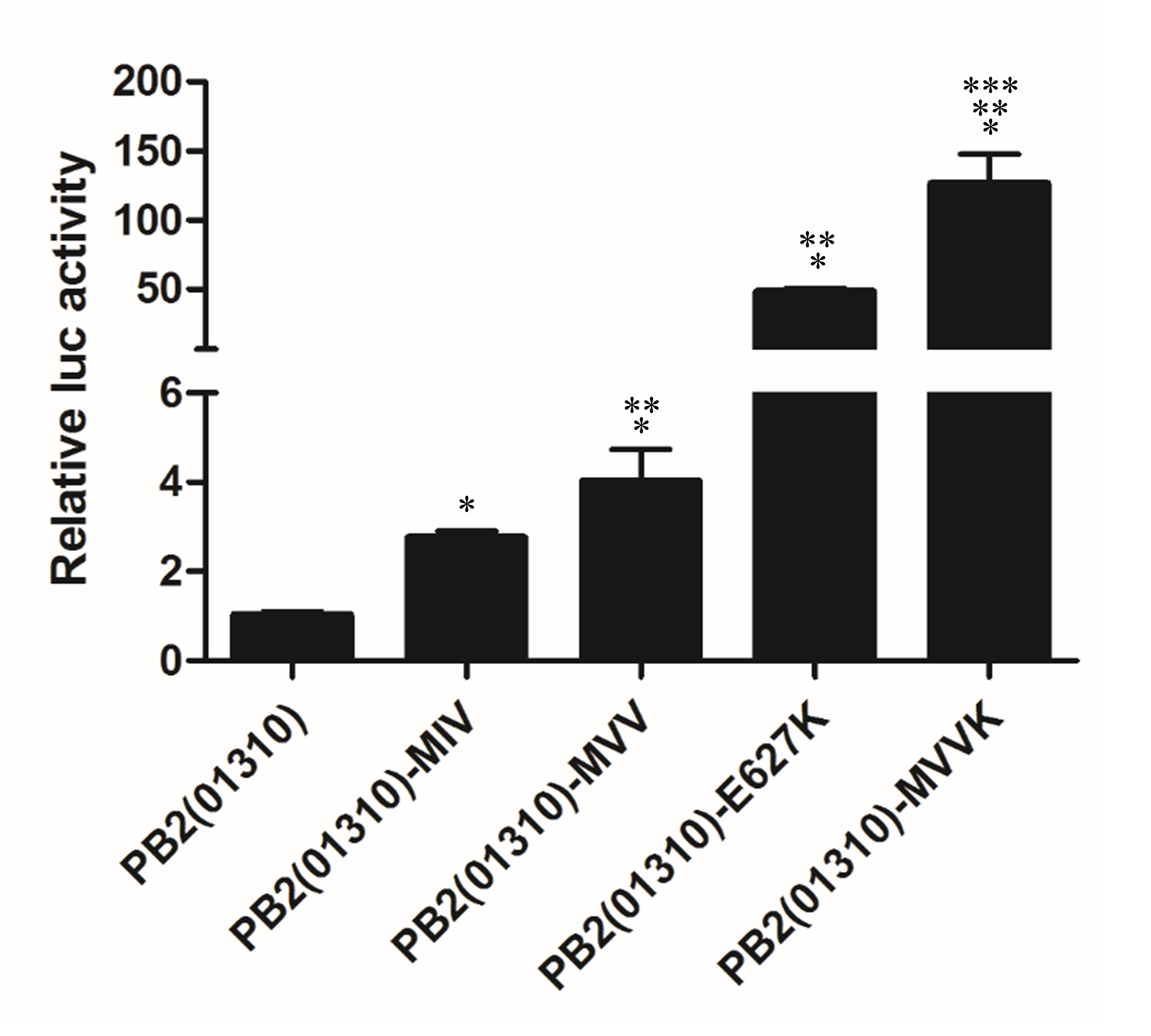
**

Viral polymerase activities of 01310 PB2 variants were measured using mini-genome assays in 293T cells at 33°C. The data were normalized to the polymerase activity of the wild-type 01310 PB2 gene. Statistical significance was calculated using Student’s t-test (compared to PB2(01310), ^*^*P* < 0.05; compared to PB2(01310)-MIV, ^**^*P* < 0.05; compared to PB2(01310)-E627K, ^***^*P* < 0.05).

**Figure S2. Replication efficiency of 01310 PB2 variants in porcine and human cell lines.**

**
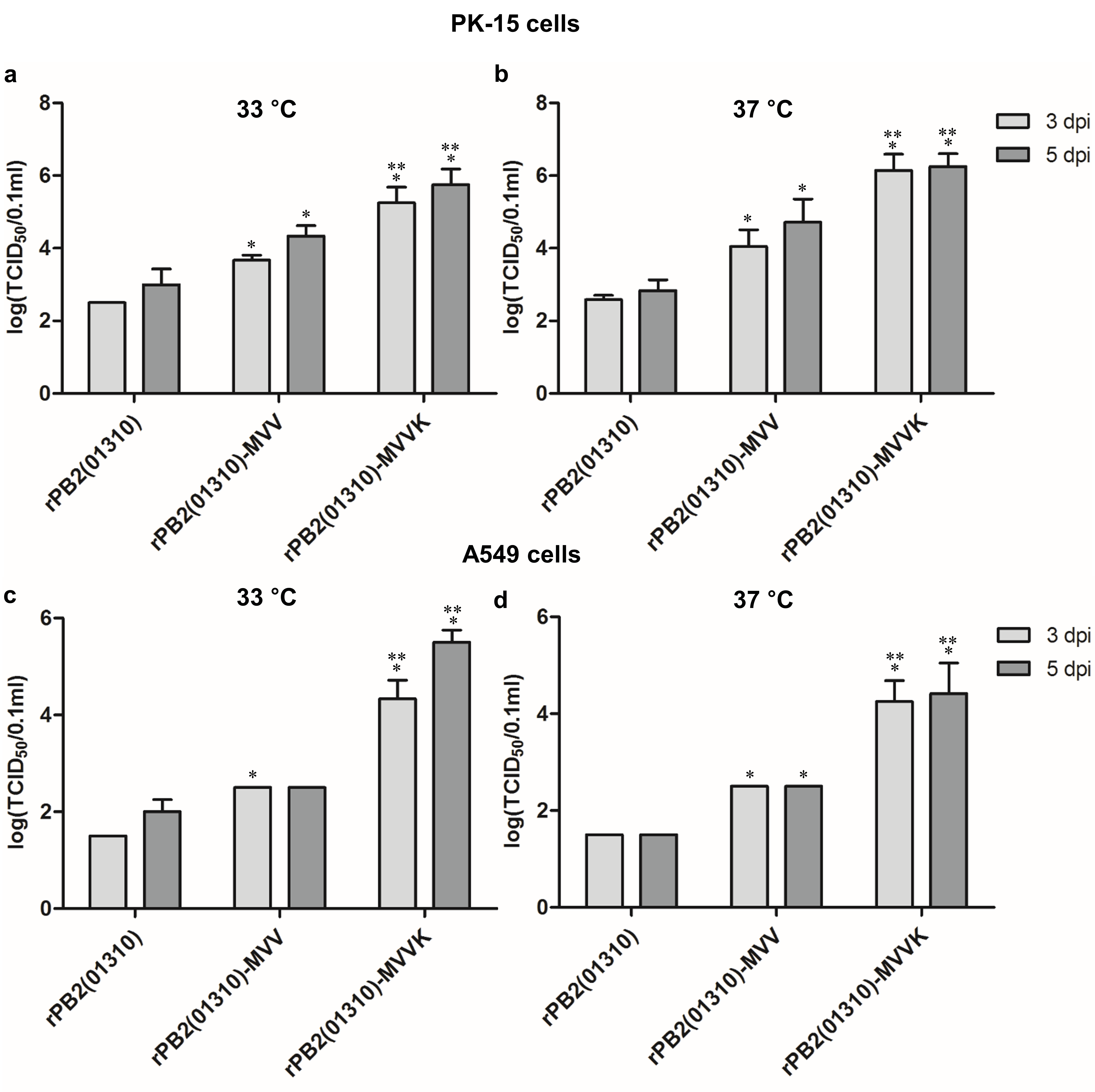
**

Replication efficiency of rPB2(01310), rPB2(01310)-MVV, and rPB2(01310)-MVVK in PK-15 (**a**,**b**) and A549 (**c**,**d**) cells at 33°C (**a**,**c**) and 37°C (**b**,**d**). Wild-type rPB2(01310), rPB2(01310)-MVV, and rPB2(01310)-MVVK were infected to PK-15 and A549 cells at 10^7^ EID_50_/0.1ml, and the TCID_50_ was measured at 3 and 5 dpi. Statistical significance was analysed by one-way analysis of variance with Bonferroni post-test correction (compared to rPB2(01310), ^*^*P* < 0.05; compared to rPB2(01310)-MVV, ^**^*P* < 0.05). The data presented are the average ± s.d. of three independent experiments.

**Figure S3. Histopathology of the lung tissue of the BALB/c mice infected with PR8 viruses with mutated PB2.**

**
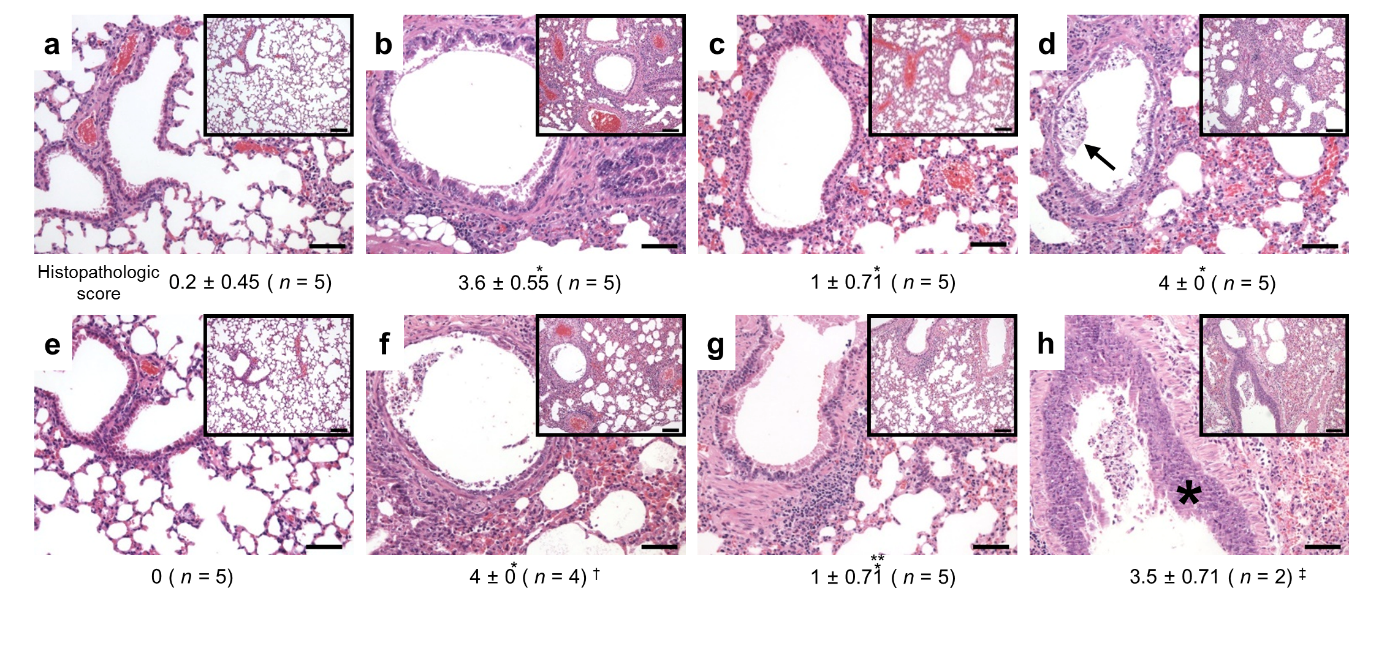
**

The lung samples of each group of mice inoculated intra-nasally with 10^6^ EID_50_/50 µl of PB2 mutated PR8 viruses or PBS were collected at 6 days post inoculation. rPB2 (01310)-E627K (**b**) and rPB2(01310)-MVVK (**d**) induced more severe inflammation than rPB2(01310) (**a**). **r**PB2(01310)-E627K and rPB2(01310)-MVVK infected mice showed necrotizing bronchiolitis and interstitial pneumonia. In particular, rPB2(01310)-MVVK cause marked bronchiolar epithelial necrosis (arrow). rPB2(E01310)-MVV (**c**) caused mild to moderate peribronchiolitis, but rPB2(01310) infected mice showed no significant histopathological lesions compared to the control mice (mock) (**e**). The pulmonary lesions of rPB2(PR8)-III infected mice (**f**) were similar to those of rPR8 infected mice (**h**), but marked proliferation of bronchiolar epithelium (asterisk) was observed in the rPR8 infected mice. rPB2(PR8)-IIIE (**g**) induced less severe inflammation compared to rPB2(PR8)-III and rPR8 (H&E, 200 x; Inset = 100 x). The histopathologic score of each group is shown in bottom of each figure. Statistical significance was analysed using the Mann-Whitney test and compared with the histopathologic score of mock ^*^*P* < 0.05 or the histopathologic score of rPR8, ^**^*P* < 0.05,

† 4 mice were measured due to 1 mouse death

‡ 2 mice were measured due to 3 mouse deaths

**Supplementary Tables and Footnotes**

**Table S1. Adaptive mutations of rPB2(01310)-MVV in 6 dpi mouse lung.**

| **Residue^†^** | **Mouse-A^*^** | **Mouse-B** | **Mouse-C** | **Mouse-D** | **Mouse-E** |
| --- | --- | --- | --- | --- | --- |
| 590S | 0/10 | 0/10 | 0/10 | 0/10 | 0/10 |
| 591R | 0/10 | **1/10** | 0/10 | 0/10 | 0/10 |
| 591K | 0/10 | 0/10 | **5/10** | 0/10 | 0/10 |
| 627K | **2/10** | **10/10** | 0/10 | 0/10 | 0/10 |
| 701N | 0/10 | 0/10 | 0/10 | **8/10** | 0/10 |

*Mouse lungs infected with rPB2(01310)-MVV were collected at 6 days post-inoculation., RT-PCR and cloning procedures were performed, and we then sequenced the indicated numbers of PB2 clones. Mammalian pathogenic factors in 590/591, 627, and 701 residues were found in lungs of mice infected with rPB2(01310)-MVV.

†Widely known important mammalian pathogenicity-associated residues are shown.

Boldface letters indicate PB2 clones with mammalian pathogenicity mutations.

Table S2. Frequencies of neighbouring amino acid residues of 66, 109 and 133 amino acid residues of PB2.

| **Segment** | **Residue** | **Host species** | | | |
| --- | --- | --- | --- | --- | --- |
|  |  | **Birds** | **Pigs** | **Humans**  **(H5/H6/H7/H9/H10)** | **Humans**  **(H1/H2/H3)** |
| PB1 | 613 | W (99.99) | W (99.93) | W (100) | W (100) |
|  | 621 | Q (95.86)  R (2.55)  K (1.58) | Q (10.06)  R (89.30)  K (0.64) | Q (100) | Q (12.12)  R (87.86)  K (0.01) |
|  | 628 | L (88.70)  M (10.77) | L (98.20)  M (0.57) | L (68.86)  M (30.80) | L (99.93)  M (0.01) |
|  | 629 | N (99.99) | N (99.95) | N (100) | N (99.99) |
|  | 630 | P (99.99) | P (100) | P (100) | P (99.99) |
| PA | 429 (434) ^*^ | P (99.97) | P (100) | P (100) | P (99.99) |
|  | 430 (435) | I (99.94) | I (99.76) | I (100) | I (99.99) |
|  | 433 (438) | I (99.62) | I (98.49) | I (99.67) | I (99.79) |

*numbering based on PA of IAVs from birds, pigs and humans.
